# Novel Adsorbent Material from *Plinia cauliflora* for Removal of Cationic Dye from Aqueous Solution

**DOI:** 10.3390/molecules28104066

**Published:** 2023-05-12

**Authors:** Natalia Nara Janner, Luana Vaz Tholozan, Guilherme Kurz Maron, Neftali Lenin Villarreal Carreno, Alaor Valério Filho, Gabriela Silveira da Rosa

**Affiliations:** 1 Chemical Engineering, Federal University of Pampa, Bagé 96413-172, Brazil; 2 Graduate Program in Materials Science and Engineering, Technology Development Center, Federal University of Pelotas, Pelotas 96010-610, Brazilalaorvf@msn.com (A.V.F.)

**Keywords:** adsorption, liquid effluents, kinetic, adsorption isotherm, *Plinia cauliflora*

## Abstract

The food industry is responsible for the generation of large amounts of organic residues, which can lead to negative environmental and economic impacts when incorrectly disposed of. The jaboticaba peel is an example of organic waste, widely used in industry due to its organoleptic characteristcs. In this study, residues collected during the extraction of bioactive compounds from jaboticaba bark (JB) were chemically activated with H_3_PO_4_ and NaOH and used to develop a low-cost adsorbent material for the removal of the cationic dye methylene blue (MB). For all adsorbents, the batch tests were carried out with the adsorbent dosage of 0.5 g L^−1^ and neutral pH, previously determined by 2^2^ factorial design. In the kinetics tests, JB and JB-NaOH presented a fast adsorption rate, reaching equilibrium in 30 min. For JB-H_3_PO_4_, the equilibrium was reached in 60 min. JB equilibrium data were best represented by the Langmuir model and JB-NaOH and JB-H_3_PO_4_ data by the Freundlich model. The maximum adsorption capacities from JB, JB-NaOH, and JB-H_3_PO_4_ were 305.81 mg g^−1^, 241.10 mg g^−1^, and 122.72 mg g^−1^, respectively. The results indicate that chemical activations promoted an increase in the volume of large pores but interacted with functional groups responsible for MB adsorption. Therefore, JB has the highest adsorption capacity, thus presenting as a low-cost and sustainable alternative to add value to the product, and it also contributes to water decontamination studies, resulting in a zero-waste approach.

## 1. Introduction

Food industry production has been growing at a very fast pace, which can be related to population and urbanization growth, leading to concerns regarding the management of the residues produced by this sector [1,2]. Thus, the negative environmental, economic, and social impacts caused by food waste have been increasing over the years. To overcome this scenario, numerous studies involving the reuse of these wastes as value-added compounds have been recently reported [3,4,5,6,7].

Jaboticaba (*Plinia cauliflora*) is a Brazilian fruit originating from the Atlantic Forest that presents spherical and black-colored peels and is naturally adapted to adhere to the stem of the jaboticaba tree. The jaboticaba peel has a high concentration of phenolic compounds, which is responsible for the antimicrobial and antioxidant properties of the fruit [8]. Due to its organoleptic characteristics and high productivity, jaboticaba is used in the food industry for the production of jellies, sweets, and beverages [9,10].

The industrial processing of these products generates high amounts of residues, such as peels and seeds, which need to be treated and correctly disposed to prevent potential environmental impacts, resulting in costs for the industries. There are several reports describing the reuse of agro-industrial residues for the production of natural extracts. However, in most cases, the solid residues generated during the extraction processes are discarded, rejecting lignocellulosic materials with a high potential for the production of bioadsorbents. As a promising alternative, the conversion of wastes into valued-added materials has been studied for numerous applications, such as activated carbons for adsorption, biochars, and active compounds for food packaging [5,11,12,13,14,15]. Thus, recent studies have been investigating the use of industrial residues for the production of new adsorbent materials as an alternative to commercial activated carbon (AC), considering that AC is responsible for 70% of the costs involved in the adsorption operation due to the high energy consumption needed during thermal treatment [16,17]. 

The production of low-cost bioadsorbents can be carried out through chemical or physical activation. The chemical activation is usually cheaper and is normally based on processes that employs a dehydrating agent followed by neutralization, resulting in a porous material with high specific surface area [18]. The chemical activation can be carried out using various reagents, including H_3_PO_4_—which is preferred due to environmental and economic aspects as well as its ability to develop macro- and mesopores in the adsorbent [19], although it can improve the ash content of the material developed—and NaOH, which is widely used due to its low cost and capacity to produce high surface area adsorbents [20,21]. Accordingly, it is reported the use of organic wastes as environmentally friendly, sustainable, and renewable sources for the development of bioadsorbents applied in the removal of contaminants from aqueous solutions, such as golden trumpet tree bark, mulberry branches, and oxidized chitosans [22,23,24]. However, the reuse of jaboticaba peels as an alternative source has not been reported.

Since water decontamination has been widely investigated due to the increase in pollution by industrial activities, the use of the residues generated in the extraction of jaboticaba peels for the production of a bioadsorbent for treatment of aqueous effluents can be an interesting alternative for the valorization of this biomass, as it purposes the reuse of a residue generated by a product that was already developed using a residue as raw material—the jaboticaba peel [24]. The textile industry is responsible for a high percentage of this pollution, discarding high amounts of pigments in watercourses [11,25]. One of the most popular textile contaminants is the cationic dye methylene blue (MB) whose process of degradation is complex and has high toxicity [26]. Among the processes used in the removal of contaminants from effluents, adsorption is one of the most efficient [25,27,28,29], comprising a surface phenomenon that consists in transferring the contaminants from an aqueous solution to a solid phase. Besides high efficiency, this process is also considered excellent due to its low cost, availability, profitability, and ease of operation [30].

Herein, we describe for the first time an investigation involving the reuse of the solid waste produced in the process of extraction of phytochemical compounds from the jaboticaba peels, which is also considered a residue, as novel adsorbents of MB from simulated textile effluents, revealing a zero-waste approach. Additionally, the purpose of this work brings the development of materials with a cheaper preparation process and higher adsorption capacity when compared to materials with complex and expensive syntheses [31,32]. The adsorbents were characterized through scanning electron microscopy, thermogravimetric analysis, X-ray diffraction, and Fourier-transform infrared spectroscopy. The adsorbent–MB interaction was evaluated through experimental design and kinetic and isothermal models. 

## 2. Results and Discussion

### 2.1. Characterization of JB, JB-H_3_PO_4_, and JB-NaOH Bioadsorbents

**Figure 1:**
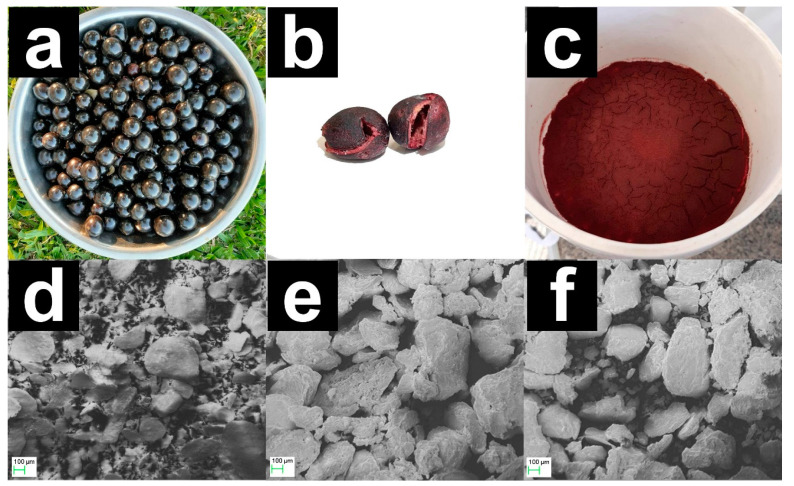
Post-harvest jaboticaba (**a**); dried jaboticaba bark (**b**); jaboticaba extract residue (**c**); and SEM micrographs for JB (**d**), JB-H_3_PO_4_ (**e**), and JB-NaOH (**f**).

Figure 1a–c illustrate the sequence of modification of the raw material, represented by the jaboticaba fruit after harvest (a), the dried JB (b), and the residue generated in the extraction of biocompounds (c) [11]. During the extraction of phenolic compounds from the JB, there is a break between the bonds of different functional groups, leaving the surface of the bioadsorbent more susceptible to interaction with MB molecules [11]. The SEM images of JB, JB-H_3_PO_4_, and JB-NaOH are exhibited in Figure 1d–f, respectively, indicating that the three samples can be classified as a fine particulate material with irregular and heterogeneous surfaces, exhibiting cavities and protuberances. Moreover, the JB-H_3_PO_4_ and JB-NaOH samples presented larger cavities when compared to JB due to the chemical activation with NaOH and H_3_PO_4_, which results in the degradation of components such as lignin and hemicellulose [6,33]. These characteristics agree with previous studies that characterized adsorbents from lignocellulosic wastes [34]. 

Table 1 describes the results of the physical properties of JB, JB-H_3_PO_4_, and JB-NaOH. The data were subjected to the Tukey test at a significance level of 0.05, and there are no significant differences between the three adsorbents. The values of *ρ_b_*, *ρ_r_*, and *ε* are in accordance with the study described by Costa et al. (2015), which characterized açai pulp and obtained the value of 1.02 g cm^−3^ for real density [35].

**Figure 2:**
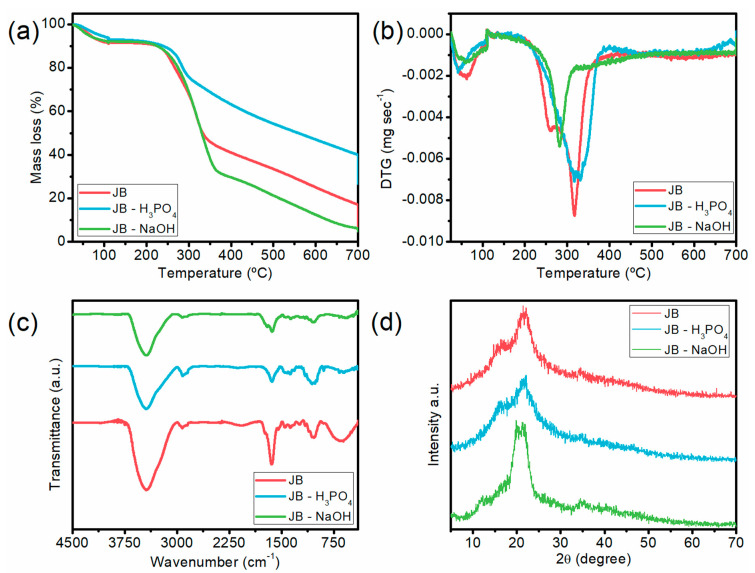
Characterization JB, JB-H_3_PO_4_, and JB-NaOH. Thermogravimetric (**a**), thermogravimetric derivative (**b**), FTIR spectra (**c**), and XRD pattern (**d**).

Thermogravimetric analysis (TGA) was performed to evaluate the thermal stability of the samples, and the results are shown in Figure 2a. The TGA curves for all samples show four well-defined stages, and an initial weight loss at temperatures lower than 100 °C was observed for all samples due to the evaporation of moisture and low molecular compounds, representing less than 10% of the mass loss [36]. The DTG curves (Figure 2b) show peaks at approximately 280 °C for JB-H_3_PO_4_ and near to 320 °C for JB and JB-NaOH, which represents the simultaneous degradation of lignin (100–900 °C), hemicellulose (235–315 °C), and cellulose (315–370 °C) [37]. The degradation observed after 350 °C is characterized by the absence of peaks, which indicates the constant degradation of lignin [38].

In order to identify the functional groups attached to the surface of the samples, FTIR measurements were performed, and the spectrum recorded for JB, JB-H_3_PO_4_, and JB-NaOH are displayed in Figure 2c. All samples presented an intense band between 3600 and 3400 cm^−1^, which is attributed to the presence of alcoholic and phenolic groups (O-H) [30,39,40]. The slight bands around 2900 cm^−1^ are related to sp^2^ C-H from cellulose and sp^3^ C-H stretching from hemicellulose. The bands in the range of 1400–1700 cm^−1^ represent non-conjugated C=O stretching of hemicellulose structure [41], and the peaks at 1050 cm^−1^ are related to C-O-C of the β–glycosidic linkages and represent the stretch of lignocellulosic compounds [42]. The peak at 672 cm^−1^ observed in JB spectra is related to C-H stretching [38]. The FTIR spectra show a decrease in the peaks of O-H groups after activation, which can be related to the hydroxylation in the surface for the basic treatment. The JB-H_3_PO_4_ showed a decrease in the O-H peak and the disappearance of the C-H peak, related to the dehydration promoted by the treatment [43]. The modification in the bands indicates that the activation was able to modify chemical characteristics of the adsorbents. Furthermore, functional groups on the bioadsorbent surface often play a major role in the adsorption of various contaminants [26]. The main mechanism of MB adsorption is based on the electrostatic interaction between the dye molecules and the negatively charged C=O group. Additionally, the π–π interaction between the aromatic rings of MB molecule and the adsorbent can be considered a mechanism of MB adsorption [44]. The presence of these functional groups is considered very important in the adsorption of dyes, as they promote anchorage points that interact by electrostatic means [45]. 

The crystal structure of the materials was characterized by X-ray diffraction (XRD), and the XRD patterns of JB, JB-H_3_PO_4_, and JB-NaOH are shown in Figure 2d. The characteristic diffractograms of lignocellulosic materials with peaks in the region of 10–25° can be observed for the three samples [46]. Thus, a decrease in the cellulose peak for JB-H_3_PO_4_ when compared to JB was noticed, indicating that the acid treatment provided a disturbance in the crystal structure of the material, promoting the breakage of crystal chains and the widening of the amorphous structure. It suggests that a fraction of the crystalline cellulose was converted into amorphous cellulose [47]. The increase in the intensity of the peak at 20.13° for JB-NaOH can be attributed to the alkaline treatment, causing an increase in the crystallinity of the fibers due to the hydrolyzation of the amorphous regions during the NaOH treatment [48]. The absence of other peaks confirms that the three samples have a predominantly amorphous structure, which may favor the adsorption of methylene blue dye [26,49,50].

### 2.2. Factorial Design

**Figure 3:**
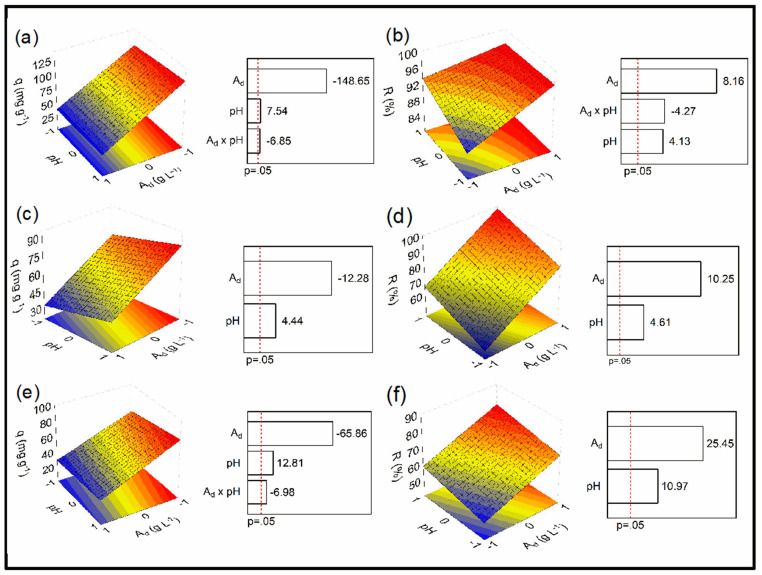
Response surface and Pareto charts for adsorption capacity and percent removal of JB (**a**,**b**), JB-H_3_PO_4_ (**c**,**d**), and JB-NaOH (**e**,**f**).

A 2^2^ factorial design was used to determine the optimized conditions for the adsorption of MB onto the adsorbents developed in this study. The conditions and results of the experimental design are shown in Table 2 and Figure 3, as well as the values of A_d_ (g L^−1^) and pH. The three samples exhibited that satisfactory results in terms of q and R values. The adsorbent that provided the best results was JB, followed by JB-H_3_PO_4_ and JB-NaOH. The tests performed with the JB reached a q value greater than 125 mg g^−1^ (run 3) and removal percentages above 96% (runs 6 and 7). The JB-H_3_PO_4_ reached a q of 90 mg g^−1^ (run 3) and a removal percentage greater than 97% (run 4), while the JB-NaOH presented an adsorption capacity of approximately 80 mg g^−1^ (run 3) and a 77% removal percentage (run 4). The Pareto charts showed that A_d_ and pH variables presented significant effects on both responses for the three samples, as they are located at the right side of the significance level (*p* ≤ 0.05). The interaction between the parameters had a significant effect only on the adsorption capacity of JB and JB-NaOH as well as in the removal percentage of JB. The adsorbent dosage was the parameter that had the greatest effects on the responses, and this effect was negative for the adsorption capacity for all adsorbents, which means that, the lower the adsorbent dosage, the greater the adsorption capacity. This relation can be explained by the number of available sites in each case [51,52]. When the A_d_ is low, there are fewer free sites, which results in a high adsorption capacity. On the other hand, when the dosage is high, there is a high number of free sites available, promoting a greater removal percentage [30]. Furthermore, a high adsorption dosage can cause aggregation of particles and reduce the adsorbent surface area.

For removal percentage, the adsorbent dosage had a positive effect, indicating that the greater the adsorbent dosage, the greater the removal percentage obtained, since at higher adsorption dosages, there will be more available sites and the accessibility to these pores become easier [53]. The pH had a positive effect on all responses, which can be attributed to the fact that MB is a cationic dye. This parameter has great importance to the adsorption process of dyes because it controls the intensity of electrostatic forces of charges provided by the dye molecule [54]. At a pH lower than 5, the H^+^ molecules of the dye will repulse the negatively charged adsorbent surface, which results in the protonation of the functional groups located at the adsorbent surface and, consequently, reduces the removal efficiency [55,56]. The high pH increases the electrostatic forces that are responsible for the attraction of the anions attached on the solid surface and the cations that form the dye molecules [57]. Furthermore, the removal of this dye is also favored in basic solutions, as at low pH, there is a high amount of positive ions in the solution, which can compete with the MB positive ions by occupying the available sites on the surface of the adsorbent [58]. After investigating the pH effects of the MB solutions on *q* and *R* values, it was selected to maintain the neutral pH of the MB solutions, since the effluents contaminated with MB are normally found in a neutral pH.

The ANOVA analyses (Table S2) showed a regression significance, which is confirmed by the F_value_ > F_tabled_ for both parameters for all adsorbents. These values are considered satisfactory, indicating that the models can predict the adsorption capacity and removal efficiency responses for the conditions used in this study. Equations (1)–(6) describe the dependent variables q and R for JB, JB-NaOH, and JB-H_3_PO_4_, respectively.

(1) $$q=76.2058-38.6597A_{d}+1.9602\mathrm{pH}-1.7804A_{d}\mathrm{pH}+0.5411$$

(2) $$q=50.2224-20.1008A_{d}+3.9109\mathrm{pH}-2.1300A_{d}\mathrm{pH}+0.7451$$

(3) $$q=57.7496-18.9842A_{d}+6.8587\mathrm{pH}-19.1100$$

(4) $$R=93.2779-3.2894A_{d}+1.6662\mathrm{pH}-1.7236A_{d}\mathrm{pH}+1.3014$$

(5) $$R=64.0535-9.5716A_{d}+4.1260\mathrm{pH}-1.1318$$

(6) $$R=74.7555-16.4647A_{d}+7.4046\mathrm{pH}-20.6382$$

### 2.3. Kinetic and Equilibrium Studies 

**Figure 4:**
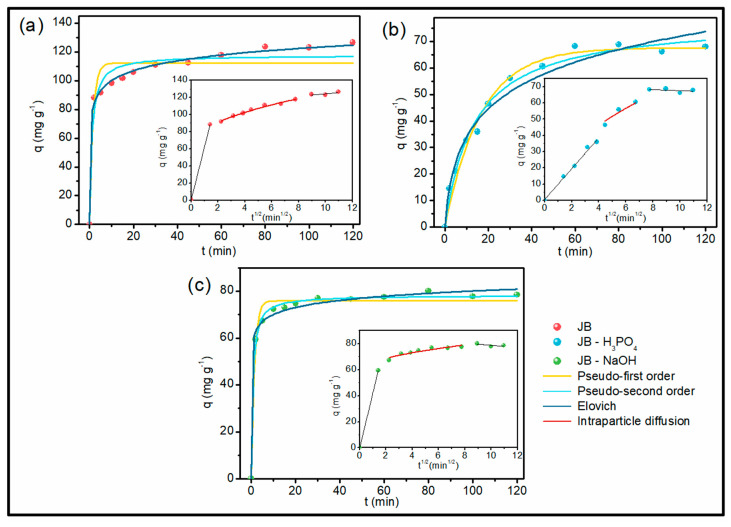
Kinetic models for JB (**a**), JB-H_3_PO_4_ (**b**), and JB-NaOH (**c**); volume of dye solution = 25 mL; temperature = 25 °C; adsorbent dosage 0.5 mg; pH = 7; agitation speed = 150 rpm.

Figure 4 illustrates the results obtained in the kinetic experiments of MB removal through the bioadsorbents. JB (Figure 4a) and JB-NaOH (Figure 4c) showed faster adsorption, while JB-H_3_PO_4_ (Figure 4b) presented a slightly slower adsorption. In total, 80% of MB was adsorbed by JB within 30 min, reaching equilibrium with approximately 90% of removal after 60 min. The JB-NaOH removed about 50% in the first 15 min of adsorption, reaching equilibrium at 30 min with 57% removal. Finally, JB-H_3_PO_4_ adsorbed approximately 40% of the dye in the first 30 min, reaching equilibrium with 50% removal in 60 min. Royer et al. (2009) used a Brazilian pine fruit shell for the production of an adsorbent material applied in the removal of MB, reaching equilibrium in 240 min [59]. Jawad et al. (2018) performed MB removal studies in adsorbent materials obtained from pomegranate (*Punica granatum*) peels, in which the equilibrium was reached in 120 min [60]. These results indicate that the studies carried out with JB, JB-NaOH, and JB-H_3_PO_4_ showed good adsorbent–adsorbate interaction, requiring a shorter time for the contaminant removal. The values obtained in the adjustments of the adsorption kinetic models for the three samples are presented in Table 3.

The fitting for JB occurred for the Elovich model, indicated by the highest value of R^2^ and lowest values of ARE and *ꭕ*^2^. This model indicates that the adsorption decreases with the increasing adsorbent–adsorbate contact time, which is attributed to the coverage of the adsorbent surface by the contaminant [61]. For JB-NaOH, the best fit occurred for the pseudo-second-order model, suggesting that the mechanism occurred was the chemisorption, characterized by a chemical reaction between the adsorbent and adsorbate, which also agrees with the good fit for the Elovich model [62,63]. The pseudo-first- and pseudo-second-order models presented a good fit for JB-H_3_PO_4_. The pseudo-first-order model indicates that van der Waals forces and H bonding can occur in the MB adsorption [64,65]. The intraparticle diffusion model is used to indicate the limiting steps of adsorption. The first step indicates the diffusion of the contaminant onto the adsorbent surface, while the intraparticle diffusion occurs at the second step. The third and last step represent the equilibrium plateau region, where the ions of the contaminant use all the available sites of the adsorbent [66]. Figure 4 shows that, for all materials, the linear portion of intra-particle diffusion data did not pass through the origin, which represents a variation of mass transfer in the initial ad final stages of the adsorption process. This indicates that the adsorption of MB onto JB, JB-H_3_PO_4_, and JB-NaOH was a multi-step process with adsorption on the external surface and diffusion into the interior [67].

**Figure 5:**
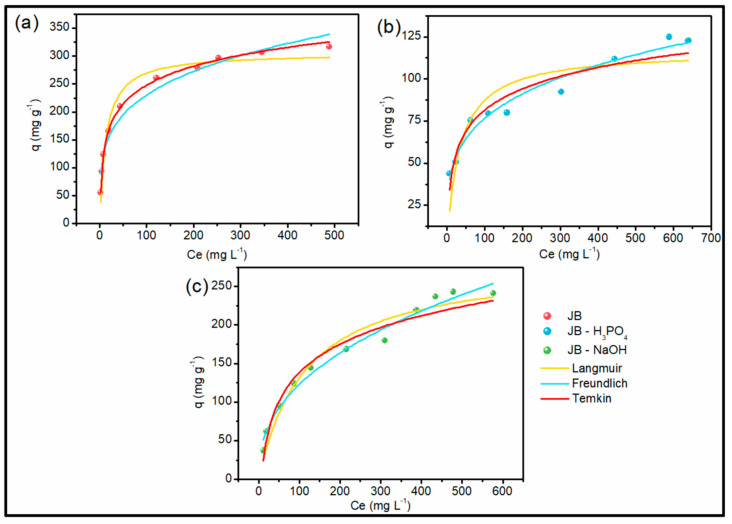
Results of the equilibrium experiments for JB (**a**), JB-H_3_PO_4_ (**b**), and JB-NaOH (**c**) volume of dye solution = 25 mL; temperature = 25 °C; adsorbent dosage 0.5 mg; pH = 7; agitation speed = 150 rpm.

According to Figure 5, all adsorbent materials presented favorable isotherms, and the convex format indicates that low concentrations of adsorbate promoted high adsorption [68]. The isotherms obtained are classified as type IV and V, representing a monolayer adsorption process, which is common on microporous materials [14,69]. Table 4 presents the values of the parameters obtained in the adjustment of the equilibrium isotherm models.

The results show that the best fit for JB occurred in the Temkin model, which was the one that presented the higher values of R^2^ and lower values of ARE and X^2^. This model indicates that the heat during the adsorption decreases linearly during the process due to the adsorbent–adsorbate interaction. The values of A_T_ and *B* represent the binding energy and adsorption heat, respectively. It is possible to observe that the value of B was positive, which indicates that the adsorption process was exothermic [70]. Additionally, this model is an indication that the molecules of adsorbate are attracted on the surface with strong intermolecular attraction [71]. Consequently, the adsorption stops when the absorbent surface saturates [71,72]. The *k_L_* parameter of 0.07 L mg^−1^ obtained for JB is related to the free energy of adsorption, i.e., the affinity between the adsorbent and adsorbate. 

JB-H_3_PO_4_ and JB-NaOH had a better fit to the Freundlich model, indicating that the adsorbent–adsorbate interaction is heterogeneous, which promotes an exponential distribution of free energy of adsorption. Thus, it is assumed that the adsorption occurs infinitely with the increase in the concentration of adsorbate in the solution, with no equilibrium plateau (characteristic of Langmuir model) [72,73]. In this regard, there is no q_max_ parameter in this model. For comparison with the literature, the maximum experimental values of 122.72 mg g^−1^ and 241.10 mg g^−1^ for JB-H_3_PO_4_ and JB-NaOH are going to be used, respectively.

Compared to the literature, the residue from the extraction of the bioactive compounds from jaboticaba bark presents satisfactory results when used in the removal of MB. Table 5 presents a comparison of the maximum adsorption capacity of various similar adsorbents for MB. These results confirm the high potential of the proposed methodology to produce an alternative MB adsorbent material from residues. Comparing the q_max_ values of JB, JB-NaOH, and JB-H_3_PO_4_, the best result was obtained with JB, which was not submitted to chemical activation, reaching a q_max_ of 314 mg g^−1^. The high value of q_max_ obtained for JB can be related to the origin of the raw material, where the extraction might have caused an increase in the porosity of the material [74]. The lower values for JB-NaOH and JB-H_3_PO_4_ could be attributed to a deterioration of the structure caused by the chemical activation [75]. This result can be considered very promising, considering that the activation process has some disadvantages such as corrosion of the equipment, high cost of reagents, and difficult recovery of chemicals [76]. Additionally, the materials developed in this study present characteristics of catalysts, which are also used in water decontamination [77]. In this sense, the process investigated in the present study might be related to a catalysis, since this process consists of the migration of the contaminant molecules to the surface of the catalyst. When the contaminant molecule is bigger than the pore diameter, the migration occurs through intra-particle diffusion [78]. In addition, the low cost of the production of JB generates a smaller environmental impact by reducing the release of chemicals in the environment and avoiding the washing step, which requires a large amount of water and is commonly needed in chemical activation.

### 2.4. Adsorption Mechanism

**Figure 6:**
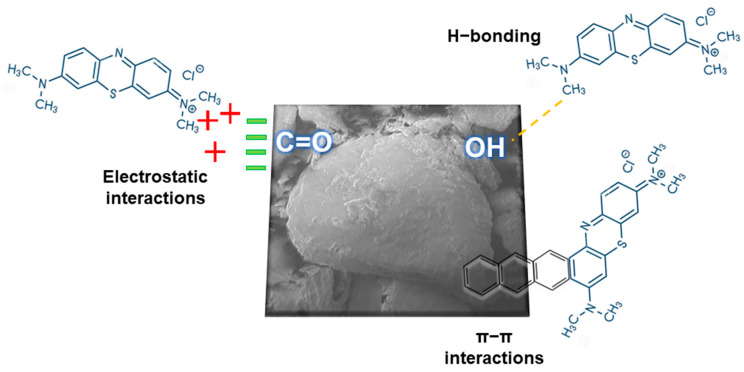
Proposed interaction of MB with the JB adsorbent.

The adsorption process is very complex and involves multiple mechanisms. These mechanisms are important to understand the basic principles of the adsorption process. The possible interactions that occur between the MB molecules (cationic dye) and JB, JB-H_3_PO_4_, and JB-NaOH can be of three types: H bonding, π–π bonds, and electrostatic interactions with the functional groups attached on the surface of the adsorbents [87]. Figure 6 presents a scheme of these interactions. Electrostatic interactions can occur by the attraction between the cations of the MB and the surface of the adsorbent when the adsorbent has a negative charge. The π–π interactions can occur due to the presence of the aromatic rings of lignin in the structures of the adsorbents and MB.

From the FTIR analysis, some functional groups present in the adsorbents (-OH and C=O) were found to be derived from the main biomass components (cellulose, hemicellulose, and lignin). These groups may have interacted by hydrogen bonds as well as Van der Waals forces [87,88]. Among the functional groups, JB has the highest amount of the OH and C=O groups on the surface. The interaction of the OH group by hydrogen bonding between the aromatic N of MB probably aimed for a higher result for q_max_. From JB-NaOH and JB-H_3_PO_4_, the analysis presented in Figure 2, Figure 3 and Figure 4 suggests that the chemical activation caused a reorganization in the JB structure, degrading the smaller pores that had been formed in the extraction of the phenolic compounds, thus forming larger pores [89]. Additionally, the chemical activation interacted with functional groups that could aid in MB adsorption [75].

## 3. Materials and Methods

### 3.1. Materials

The solid residues from jaboticaba bark were obtained from a study involving the extraction of bioactive compounds [11]. Prior to the chemical activation process, the residues were dried at 105 °C for 24 h, and here they are entitled JB. All reagents used were purchased from Sigma-Aldrich (Burlington, MA, USA). 

### 3.2. Chemical Activation

JB residues were chemically activated with phosphoric acid (H_3_PO_4_) and sodium hydroxide (NaOH). The activation with phosphoric acid was performed according to the methodology proposed by Portinho et al. (2017) [6]. In a typical experiment, 10 g of dry biomass was mixed with the H_3_PO_4_ (85% *w/w*) in a 4:1 ratio. The mixture was agitated on a shaker at 100 rpm for 24 h at room temperature. For the activation with NaOH, the methodology proposed by Silva et al. (2020) was applied [33]. In this procedure, 10 g of JB was soaked in a NaOH solution (20% *w/v*) at a 1:3 ratio for 24 h at room temperature. Both activated samples were then oven-dried at 105 °C for 24 h. Finally, the materials were washed with distilled water until reaching a neutral pH, dried in an oven, and sieved through an electromagnetic sieve shaker (Bertel, model 4830) to obtain the particle < 495 μm [30]. The samples are here called JB-H_3_PO_4_ (H_3_PO_4_-activated jaboticaba bark) and JB-NaOH (NaOH-activated jaboticaba bark).

### 3.3. Characterization

The real density (*ρ_r_)* values were obtained through helium pycnometer using a gas pycnometer (Quantachrome Corporation, Boynton Beach, FL, USA) at 18 psig and 19.5 °C. The values of the bulk density were obtained with the use of a graduated cylinder. The porosity of the particle bed (ε) was calculated according to Equation (7), which relates the *ρ_r_* (g cm^−3^) and *ρ_b_* (g cm^−3^).

(7) $$\varepsilon =1-\frac{\rho_{b}}{\rho_{r}}$$

The thermal decomposition of the materials was studied through thermogravimetric analysis, using a thermal analyzer (TA 60WS Shimadzu, Japan) and a thermobalance (TGA 50 Shimadzu, Kyoto, Japan). Analysis was carried out using approximately 8 mg of sample, heated from 25 °C to 700 °C with a heating rate of 10 °C min^−1^ in a 50 mL min^−1^ N_2_ atmosphere. X-ray diffraction technique (XRD, Rigaku Ultima IV diffractometer, Tokyo, Japan) with Cu-Kα radiation at a voltage of 40 kV was used to investigate the crystalline structure of the adsorbents. The XRD patterns were recorded in the 2θ range from 5° to 70°.

Fourier-transform infrared spectroscopy (FTIR) was carried out to identify the main functional groups on the surface of JB, JB-H_3_PO_4_, and JB-NaOH. The results were obtained in a Shimadzu 01722, IR Prestige, Japan spectrophotometer, using diffuse reflectance with KBr. The spectra were obtained over the range of 400–4000 cm^−1^ and a resolution of 4 cm^−1^. The morphological characteristics of the materials were examined by scanning electron microscopy (SEM, JEOL, JSM 6610 L V, Akishima, Japan) at an acceleration voltage of 5 Kv and at magnifications of 100, 300, and 500×.

### 3.4. Adsorption of MB onto JB, JB-H_3_PO_4_, and JB-NaOH

The MB concentrations in the liquid phase were quantified by a UV–VIS spectrophotometer (Kazuaki, II-226, Hangzhou, China) at 665 nm, using a standard curve with concentrations ranging from 0.5 to 50. The adsorption trials were carried out in batch, and the operating range conditions were obtained from preliminary trials. For the batch studies, 25 mL of MB solution (at a concentration of 70) was added to 0.5 g L^−1^ of adsorbent dosage. Then, the adsorbent–adsorbate mixtures were agitated in a shaker (NOVA ÉTICA, model 109-1, Minas Gerais, Brazil) for a varied time at 120 rpm and centrifuged for 10 min at 3000 rpm to separate the adsorbents from the MB solution. The best condition for adsorption capacity and percentage of MB removal onto the jaboticaba bioadsorbents was evaluated by the experimental design carried out through a 2^2^ factorial with 4 tests and 3 central points performed in duplicate. The levels and real values of the evaluated parameters are presented in Table 6. The remaining MB concentrations were quantified in a spectrophotometer UV-Vis, and the adsorption capacity (q) and removal percentage (R) were calculated according to Equations (8) and (9), respectively,

(8) $$q=\frac{C_{i}-C_{f}}{M}\;V$$

(9) $$R=\frac{C_{i}-C_{f}}{C_{i}}100$$

where C_i_ is the initial concentration (mg L^−1^), C_f_ is the final concentration (mg L^−1^), M is the mass of adsorbent (g), and V is the volume of solution (L).

Statistical analysis of experimental data was performed with the use of the 10th version of STATISTICA. The Pareto diagram was used to determine the effect and verify the significant variables for *q* and *R* responses. The adequacy of the models was analyzed through a variance analysis (ANOVA) with F-test (Fisher test). The response surface was generated from the values predicted by the models and used to define the conditions of adsorption tests. The first-order model that predicts the responses is represented by Equation (10).

(10) $$Y=\beta o+\sum_{i=1}^{k}\beta_{i}X_{i}+\sum_{i<j}^{\;}\sum \beta_{ij}X_{i}X_{j}$$

where *β_o_* is *the* constant coefficient, *β_i_* is the linear coefficient, *β_ii_* is the quadratic coefficient, *β_ij_* is the interaction coefficient, *X_i_* is the independent variable level, *k* is the number of variables, *e* is the experimental error, and *Y* is the model response.

The kinetic experiments were performed with the adsorbent dosage, MB concentration, and pH predicted by experimental design. The samples were agitated in a shaker at 150 rpm and collected at different times (2–120 min). The adsorption kinetic data obtained were fitted to pseudo-first-order [90] pseudo-second-order [91], Elovich [92], and Weber and Morris models [93]. In the adsorption equilibrium experiments, the MB solutions at different concentration ratios (30–700) were kept in contact with the adsorbent until equilibrium, previously determined by the kinetic studies. The equilibrium data were fitted to Langmuir [72] and Freundlich [73] models. Table S1 (Supplementary Materials) presents the adsorption isotherm and kinetic equations used in this study.

The kinetic and isotherm parameters were evaluated through non-linear regression. The adsorption capacities obtained in the models were analyzed based on the values of average relative error (ARE) [94] and Chi-square (X^2^) [95], represented by Equations (11) and (12), respectively. These analyses were applied as fit quality indicators.

(11) $$ARE=\frac{100}{nn}\sum \frac{\left(q_{exp}-q_{pred}\right)}{q_{exp}}$$

(12) $$X^{2}=\sum \frac{{\left(q_{exp}-q_{pred}\right)}^{2}}{nn-NN}$$

where *q_exp_* is the experimental value, *q_pred_* is the value predicted by the model, *nn* is the number of experiments observed, and *NN* is the number of parameters in the model.

## 4. Conclusions

This work described the production of a novel adsorbent material for MB from jaboticaba bark residues. The JB residues were chemically activated with H_3_PO_4_ and NaOH, aiming to evaluate their effect on the adsorption properties of the material. The results were in agreement with previous reports described in the literature for similar materials. The TGA and XRD analyses showed that the activated materials had slightly lower cellulose peaks compared to JB, indicating that chemical activation did not have a significant influence on the degradation of this compound. Thus, JB and JB-H_3_PO_4_ reached equilibrium in 60 min, whilst JB-NaOH presented the fastest rate, reaching equilibrium in 30 min. The kinetic models that best fit the experimental data for JB and JB-NaOH were the Elovich and pseudo-second-order models, respectively. JB-H_3_PO_4_ best fit to pseudo-second-order and intraparticle diffusion models. The maximum adsorption capacity values obtained for JB, JB-NaOH, and JB-H_3_PO_4_ were 305.81 mg g^−1^, 241.10 mg g^−1^, and 122.72 mg g^−1^, respectively. The Langmuir isotherm model best described the experimental data for JB. For JB-NaOH and JB-H_3_PO_4_, the best fit occurred for the Freundlich model. The best adsorption capacity was obtained with the adsorbent material without chemical treatment. This is a promising outcome, resulting in an effective, cheap, and eco-friendly product. This research reveals a zero-waste approach, since the raw material used for the fabrication of the adsorbent was a residue from the agroindustry in which the extraction of biocompounds had already been carried out, promoting the complete use of fruit.

## Figures and Tables

**Table 1:** Results of physical characterization of JB, JB-H_3_PO_4_, and JB-NaOH samples.

Parameter	JB	JB-H_3_PO_4_	JB-NaOH
*ρ_b_*	1.5133 ± 0.0012 ^a^	1.5118 ± 0.0005 ^a^	1.5613 ± 0.0018 ^a^
*ρ_r_*	0.6091 ± 0.0023 ^a^	0.6001 ± 0.0056 ^a^	0.4774 ± 0.0054 ^a^
*ε*	0.5975	0.6030	0.6942

^a^ Mean values in each row with different letters are significantly different (Tukey test, *p* < 0.05).

**Table 2:** Experimental results for adsorption capacity and removal percentage of MB.

			JB		JB-H_3_PO_4_		JB-NaOH	
	A_d_	pH	q	R	q	R	q	R
	(g L^−1^)		(mg g^−1^)	(%)	(mg g^−1^)	(%)	(mg g^−1^)	(%)
1	0.5	7	117.72 ± 0.44	84.43 ± 1.99	69.81 ± 2.10	49.89 ± 2.30	68.22 ± 1.21	49.52 ± 1.27
2	1.5	7	43.96 ± 0.18	94.45 ± 0.38	38.50 ± 2.10	82.51 ± 0.36	32.28 ± 0.17	69.17 ± 0.01
3	0.5	11	125.20 ± 1.19	91.21 ± 0.21	90.19 ± 6.26	64.39 ± 3.95	80.30 ± 0.22	58.28 ± 1.08
4	1.5	11	44.32 ± 0.28	94.34 ± 0.47	45.56 ± 0.27	97.63 ± 0.06	35.84 ± 0.24	76.91 ± 1.03
5	1.0	9	67.07 ± 0.01	95.24 ± 0.20	56.44 ± 2.33	80.14 ± 3.47	45.71 ± 0.58	65.69 ± 0.19
6	1.0	9	67.84 ± 0.26	96.72 ± 0.17	53.39 ± 4.22	76.09 ± 5.57	44.42 ± 0.16	64.22 ± 0.22
7	1.0	9	67.32 ± 0.59	96.55 ± 0.31	50.35 ± 0.67	72.65 ± 0.82	44.77 ± 0.71	64.58 ± 0.12

**Table 3:** Kinetic parameters for JB, JB-H_3_PO_4_, and JB-NaOH.

		JB	JB-H_3_PO_4_	JB-NaOH
Pseudo-first order	q_e_	112.10	67.57	75.86
	k_1_	0.66	0.06	0.71
	R^2^	0.91	0.98	0.98
	ARE	8.19	8.86	3.51
	ꭕ^2^	112.2	10.01	9.92
Pseudo-second order	q_e_	117.67	78.31	78.24
	k_2_	0.01	0.0009	0.02
	R^2^	0.96	0.98	0.99
	ARE	6.05	9.72	1.33
	ꭕ^2^	53.22	8.74	1.57
Elovich	α	24,043.25	11.07	4,056,450
	β	0.10	0.06	0.23
	R^2^	0.99	0.97	0.99
	ARE	1.58	7.34	2.20
	ꭕ^2^	4.61	16.09	3.97
Intraparticle Diffusion	k_di_	19.54	6.28	7.18
	C	63.30	19.48	58.52
	R^2^	0.99	0.97	0.99
	ARE	62.69	5.80	31.56
	ꭕ^2^	0.32	3.45	0.04

**Table 4:** Equilibrium parameters for JB, JB-H_3_PO_4_, and JB-NaOH.

		JB	JB-H_3_PO_4_	JB-NaOH
Langmuir	q_max_	305.81	116.78	283.55
	k_L_	0.07	0.03	0.01
	R^2^	0.97	0.77	0.96
	ARE	7.11	15.92	14.13
	X^2^	288.61	197.48	225.78
Freundlich	K_F_	75.47	24.39	18.19
	n_F_	4.13	4.02	2.41
	R^2^	0.96	0.96	0.98
	ARE	8.12	6.54	7.16
	X^2^	355.79	30.47	105.73
Temkin	B	48.68	18.35	53.25
	A_T_	1.63	0.84	0.13
	R^2^	0.99	0.85	0.95
	ARE	4.72	729.33	17.11
	X^2^	56.73	98.91	236.81

**Table 5:** Comparison of adsorption capacity of MB between this work and other related adsorbents.

Adsorbent	Activation	Co (mg L^−1^ )	Contact Time	Temperature (°C)	pH	A_d_ (g L^−1^)	q_max_ (mg g^−1^)	Ref.
JB	-	30–700	30 min	25	8	0.5	305.81	This work
JB-NaOH	NaOH	30–700	30 min	25	8	0.5	241.10	This work
Golden trumpet tree bark	-	25–300	25 min	55	10	0.5	232.25	[22]
*Cedrela fissilis*	-	50–300	60 min	25	8	1.0	230.06	[56]
*Anadenanthera macrocarpa*	-	50–300	60 min	35	8	1.0	227.57	[56]
Brazilian berry seeds	-	25–50	120 min	55	8	0.8	189.6	[79]
*Hibiscus cannabinus*	KOH	10–50	60 min	35	11	0.2	154	[80]
*Citrus sinensis*	-	50	24 h	35	7	1.0	147.4	[81]
Pine cone	NaOH	20–80	120 min	30	9.17	0.003	142.25	[82]
JB-H_3_PO_4_	H_3_PO_4_	30–700	60 min	25	7	0.5	122.72	This work
Fallen phoenix tree’s leaf	-	30–180	180 min	22	7	2.0	80.9	[83]
Orange tree sawdust	NaOH	40–100	180 min	20	6	1.0	78.74	[84]
Ryegrass straw	NaOH	150	60 min	25	8	20.0	67.19	[33]
Rice husk	-	10–120	48 h	32	8	0.5	40.5	[85]
Palm tree	-	70–700	240 min	70	6.3	1.0	39.5	[86]

**Table 6:** Independent variables and design levels.

Levels			
Factors	−1	0	1
A_d_ (g L^−1^)	0.5	1	1.5
pH	7	9	11

## Data Availability

Not applicable.

## Supplementary Materials

The following supporting information can be downloaded at: https://www.mdpi.com/article/10.3390/molecules28104066/s1, Table S1: Kinetics and isotherm models; Table S2 ANOVA for the factorial design.

## Sample Availability

Samples used in this study are available upon request through the corresponding author.

## Abbreviations

JB: jaboticaba bark; MB, methylene blue; AC, activated carbon; thermogravimetric analysis (TGA); thermogravimetric derivative (DTG); X-ray diffraction (XRD); H_3_PO_4_-activated jaboticaba bark (JB- H_3_PO_4_); NaOH-activated jaboticaba bark (JB-NaOH); real density (ρr); porosity of the particle bed (ε); bulk density (ρb); Fourier-transform infrared spectroscopy (FTIR); scanning electron microscopy (SEM); average relative error (ARE); Chi-square (X^2^).
