# Supplementary material for: Novel Adsorbent Material from Plinia cauliflora for Removal of Cationic Dye from Aqueous Solution
Source: Molecules. 2023 May 12;28(10):4066. doi: 10.3390/molecules28104066 (PMC10224290; doi:10.3390/molecules28104066)
Supplement: Supplementary file 1 [file molecules-28-04066-s001.zip › molecules-2325450-supplementary.pdf]

Supplementary Materials

Novel adsorbent material from jaboticaba bark (*Plinia cauliflora*): production, characterization, and application in the removal of cationic die from aqueous solution

Natalia Nara Janner [1], Luana Vaz Tholozan [1], Guilherme Kurz Maron [2], Neftali Lenin Villarreal Carreno [2], Alaor Valério Filho [2] and Gabriela Silveira da Rosa [1,*]

Table S1 Kinetics and isotherm models

	Equation	Model
Kinetics	$q_t = q_e(1 - e^{-k_1 t})$	Pseudo-first order
	$q_t = \left(\frac{{q_e}^2 k_2 t}{1 + q_e k_2 t}\right)$	Pseudo-second order
	$q_t = \frac{1}{\alpha} \ln(1 + \alpha\beta t)$	Elovich
	$q_t = k_{di} t^{\frac{1}{2}} + C$	Intraparticle diffusion
Isotherm	$q_e = \frac{Q_{max} K_L C_e}{1 + K_L C_e}$	Langmuir
	$q_e = K_F {C_e}^{\frac{1}{n_F}}$	Freundlich
	$qe = \frac{RT}{b} K_T C_e \ln()$	Temkin

Table S2 ANOVA for the factorial design.

	SS	DF	F_{value}	F_{tabled}	R^2	$R^2_{adjusted}$
**JB**						
q						
Regression	12012.67	3	49.03	3.71	0.9363	0.9996
Residual	816.75	10				
Lack of fit	811.88	1				
Pure error	4.87	9				
Total	1282.42	13				
R						
Regression	132.53	3	4.43	3.71	0.8708	0.9496
Residual	99.66	10				
Lack of fit	87.95	1				
Pure error	11.71	9				
Total	232.19	13				
**JB – H_3PO_4**						
	SS	DF	F_{value}	F_{tabled}	R^2	$R^2_{adjusted}$
q						
Regression	3259.52	2	137.61	3.98	0.8763	0.9538
Residual	460.04	11				
Lack of fit	288.05	2				
Pure error	171.99	9				
Total	3719.57	13				
R						
Regression	2607.31	2	280.32	3.98	0.9808	0.9885
Residual	104.21	11				
Lack of fit	25.00	2				
Pure error	185.74	9				
Total	2818.05	13				
**JB – NaOH**						
	SS	DF	F_{value}	F_{tabled}	R^2	$R^2_{adjusted}$
q						
Regression	3390.99	3	38.12	3.71	0.9196	0.9982
Residual	296.55	10				
Lack of fit	289.85	1				
Pure error	6.71	9				
Total	3687.54	13				
R						
Regression	869.12	2	280.32	3.98	0.9808	0.9885
Residual	17.05	11				
Lack of fit	6.87	2				
Pure error	10.19	9				
Total	886.17	13				

SS = sum of square; DF = degree of freedom.
